# Iranian surgeons’ compliance with the American Society of Health-System Pharmacists guideline: antibiotic prophylaxis in private versus teaching hospitals of Shiraz, Iran

**DOI:** 10.1186/1753-6561-5-S6-P150

**Published:** 2011-06-29

**Authors:** M Askarian, H Mahdaviazad, SM Masoompour

**Affiliations:** 1 Community Medicine, Shiraz University of Medical Sciences, Shiraz, Iran, Islamic Republic Of; 2 Internal Medicine, Shiraz University of Medical Sciences, Shiraz, Iran, Islamic Republic Of

## Introduction / objectives

Surgical site infections (SSIs) are the prevalent and grave complication following surgery, that significantly increase the length of hospital stay, morbidity and mortality, and subsequently drain healthcare system resources. To address the compliance with the American Society of Health-System Pharmacists (ASHP) guideline of prophylactic antibiotic in private hospitals in Shiraz, Iran.

## Methods

This was a cross sectional study using prospective data from April to September 2010 in entire surgical wards of all eleven private hospitals in Shiraz. We used descriptive analysis including frequencies for evaluating the results.

## Results

From April to September 2010, 365 patients from 63 surgical wards of eleven private hospitals were enrolled in our study. Prophylactic antibiotics were inappropriately given to 64.6% of patients. Twenty out of 26 patients did not receive antibiotic appropriately. In cases that need antibiotic prophylaxis with respect to ASHP guideline, antibiotic choice was concordant in 32 (25.4%) out of 126 procedures. Patients who needed to receive prophylactic antibiotic and received, the duration and initiation time of prophylaxis were concordant with the guideline in 37(29.4%) and 77(61.1%) respectively. The overall compliance with ASHP guideline was 10.13%.

## Conclusion

Our study revealed that in private hospitals in Shiraz, Iran about 90% of patients received inappropriate surgical prophylaxis. Thus practical measures to improve the implementation of guideline are urgently needed in the future.

## Disclosure of interest

None declared.

